# Pulsed-Field Ablation for Paroxysmal Atrial Fibrillation: Current Evidence and Its Potential Role as First-Line Therapy

**DOI:** 10.7759/cureus.100006

**Published:** 2025-12-24

**Authors:** Saleh Aloraini, Khalid Alghamdi, Wael Alqarawi, Nouf Alanazi

**Affiliations:** 1 Medicine, King Saud University, Riyadh, SAU; 2 Cardiology, King Saud University, Riyadh, SAU; 3 Electrophysiology, King Khalid University Hospital, Riyadh, SAU; 4 Interventional Cardiology, King Khalid University Hospital, Riyadh, SAU

**Keywords:** atrial fibrillation (af), cardiac catheter ablation, cryo-ablation, electroporation, paroxysmal atrial fibrillation, pulsed-field ablation, radiofrequency catheter ablation

## Abstract

Pulsed-field ablation (PFA) is a novel, non-thermal energy modality that specifically targets the myocardium by using irreversible electroporation. This review summarizes the available data regarding the effectiveness, safety, and procedural time for PFA versus traditional thermal ablation for paroxysmal atrial fibrillation. While recent trials have shown that PFA results in outcomes comparable to those of radiofrequency ablation and cryoablation for rhythm control, they have also demonstrated a significant reduction in collateral damage to surrounding structures such as the esophagus and phrenic nerve. Faster procedure times, along with a more favorable safety profile for PFA, could offer advantages for patients and health care providers. Since preliminary evidence indicates PFA may be used safely and effectively as a first-line ablation therapy for AF, additional long-term data and larger-scale real-world analyses are needed to evaluate PFA’s longevity and establish its role in routine clinical use. Overall, PFA offers a new direction for the development of catheter-based rhythm control therapies that are faster, safer, and more efficient than previously available options for atrial fibrillation.

## Introduction and background

Atrial fibrillation (AF) is the most prevalent sustained cardiac arrhythmia seen in practice and is an important and increasing global public health burden [1-4]. The number of people affected is projected to reach 12.1 million in the United States by 2030 and 72 million in Asia by 2050; both projections are driven by an aging population and increasing prevalence of comorbidities [1,4,5]. The clinical burden of AF is substantial: the presence of AF is associated with a 1.5 to 2-fold greater risk of mortality and is associated with a large increase in the risk of stroke, heart failure (HF), cognitive impairment, and myocardial infarction [6-8]. Such high rates of morbidity and mortality lead to an exorbitantly high economic burden, with AF-related care accounting for 1-2% of total health care expenditures globally, largely driven by AF-related complications and hospitalizations [4,5,7,8].

Since the description of AF with the electrocardiogram over a century ago [9,10], there has been a significant change in how AF is managed. For decades, the debate focused on rate control vs. rhythm control, and evidence from the AFFIRM (Atrial Fibrillation Follow-up Investigation of Rhythm Management) and RACE (Rate Control versus Electrical cardioversion) trials indicated that a rate control strategy with anticoagulation was non-inferior [3,9,10-12]. As summarized by Sardana and Doshi, this foundational evidence shaped clinical practice for many years [3]. However, new data have shifted this landscape. The landmark EAST-AFNET 4 (Early Treatment of Atrial Fibrillation for Stroke Prevention Trial) showed that an early rhythm-control strategy, implemented within a year of diagnosis, significantly decreased major adverse cardiovascular events, mortality, and stroke, compared to standard care [3,13]. Furthermore, within rhythm-control strategies, the ATTEST (Atrial Fibrillation Progression Trial) and STOP-AF First trial have shown that catheter ablation is superior to antiarrhythmic drugs (AADs) for maintenance of sinus rhythm and prevention of disease progression [3,14,15]. Thus, the 2023 American College of Cardiology (ACC)/American Heart Association (AHA) and 2024 European Society of Cardiology (ESC) guidelines have advanced catheter ablation to a Class 1 first-line strategy for a subset of patients with symptomatic paroxysmal AF while remaining agnostic to the energy source [6,7].

In this review, “first-line” refers specifically to catheter ablation used as an initial rhythm-control procedure in guideline-selected symptomatic paroxysmal AF (including treatment-naïve/AAD-naïve patients when applicable), rather than “early rhythm control” broadly (which may include AADs) or ablation only after mandatory AAD failure. To orient interpretation of the comparative studies discussed below, we use commonly reported procedural endpoints such as freedom from atrial arrhythmia recurrence at ~12 months (typically assessed after a 90-day blanking period), arrhythmia burden (particularly when continuous monitoring is used), and redo ablation rates.

The establishment of catheter ablation as a first-line option has shifted the clinical and research focus toward optimizing the procedure itself, particularly safety and efficiency. The conventional thermal energy modalities (i.e., radiofrequency and cryoablation) carry the inherent risk of non-selective thermal tissue damage to surrounding structures such as the esophagus or phrenic nerve [9]. These technologies rely on thermal lesion formation (heating or freezing) and can affect adjacent tissues through conductive energy transfer, which is influenced by local anatomy and lesion delivery. This is the landscape where pulsed-field ablation (PFA), a new energy source, enters the literature, as a non-thermal energy source that may allow greater selectivity of myocardial tissue, and therefore limit the risks of thermal ablation [3,7]. PFA delivers high-voltage electrical pulses that induce irreversible electroporation, causing cell death through membrane disruption with comparatively limited thermal effect; this relative myocardial selectivity provides a mechanistic rationale for lower rates of esophageal and phrenic nerve injury observed in contemporary trials and registries. Initial trials demonstrate acceptable safety and efficacy for PFA for pulmonary vein isolation [3]. While the application in the treatment algorithm remains unknown, there is emerging data on comparative effectiveness against traditional first-line thermal ablation strategies, with ongoing pivotal trials [3,7].

Background

The Early Era

The early treatment of AF was mainly pharmacological, with emphasis on rate control and, where possible, rhythm restoration with AADs [9,16]. The clinical recognition of AF dates to the 17th century when William Harvey (1628) described “pulse deficit” [9], and the arrhythmia was described as “ataxia of the pulse” or “pulsus irregularis perpetuus” [10]. The paradigm shift in AF diagnosis started with two inventions. The nature of diagnosis changed considerably. Mackenzie's "clinical polygraph" (1905) elucidated the lack of atrial contraction waves, and Einthoven's electrocardiogram (1906) provided the first electrical record of AF [9,10]. AADs were the first and primary line of therapy for AF rhythm control for the great majority of this evolution [9,16]. Digitalis compounds, which had been discovered in 1785, were probably the first rate control drugs [9,10]. Later, quinidine (1950s) and amiodarone or disopyramide (1970s) were used for rhythm control [9]. These drugs, which were repurposed for AF, were greatly limited by recurrence (up to 63%), toxicity on non-cardiac organs (e.g., amiodarone), and lethal pro-arrhythmia [16].

Major Change in Paradigm: From Drug Symptom Control to Catheter Ablation

A major transition that occurred in clinical practice is going from symptom control by pharmacologic means to the electrical substrate of AF. Electrical cardioversion (ECV), described by Lown et al. in 1962, permitted safe, drug-free sinus rhythm restoration, proving superiority to quinidine [9,17]. Catheter ablation first occurred in the 1980s by delivering a high-voltage direct current [9,18-20]. However, the risk of collateral injury, as well as death, warranted the shift to thermal ablation, radiofrequency (RF), and cryo ablation by the late 1980s [9,20]. It was not until 1998 that a breakthrough occurred when Haïssaguerre et al. showed that pulmonary vein (PV) triggers were the main initiators of paroxysmal AF [9,20]. From then on, research proceeded to demonstrate that pulmonary vein isolation (PVI) became the cornerstone of modern-day ablation [9,16]. Although early results showed notable recurrence (e.g., CAPLA (Catheter Ablation for Persistent Atrial Fibrillation) trial 53.6 % at 12 months in persistent AF), PVI remained superior in durability to prior approaches [9,16,21]. Nonetheless, thermal ablation puts patients at risk of collateral injury to adjacent structures such as the esophagus, phrenic nerve, or coronary arteries, due largely to excessive heat [20].

The Recent Era

Subsequent decades were affected by landmark clinical trials as well as better modification of ablation strategies. The AFFIRM, RACE, and Kumana et al.'s meta-analysis (2005) established that rate control was not inferior to rhythm control with respect to mortality and stroke, while at the same time decreasing drug-related adverse events [9-12,22]. Consequently, treatment guidelines favored rate control with anticoagulation for most of the patients with persistent AF [10]. To further improve outcomes beyond PVI, “PVI-plus” methods, which involved adding linear lesions or trying to gain control over non-pulmonary vein triggers, were explored [16]. However, the SMASH-AF meta-analysis noted only a modest 7.6 % incremental benefit [23]. There was no advantage with patient-tailored ablation as determined by complex fractionated atrial electrograms (CFAEs), MRI-determined fibrosis, or low-voltage areas as compared to PVI alone (DECAAF (The Efficacy of Delayed Enhancement-MRI-Guided Fibrosis Ablation vs Conventional Catheter Ablation of Atrial Fibrillation trial) II) [16,24]. More recently, “digital twin” forms of computational heart models have enabled patient-specific cardiac mapping and better efficacy (74.7% vs. 48.2%) in comparison to empirical pulmonary vein isolation [16].

The Introduction of Irreversible Electroporation (IRE)/PFA

**Figure 1:**
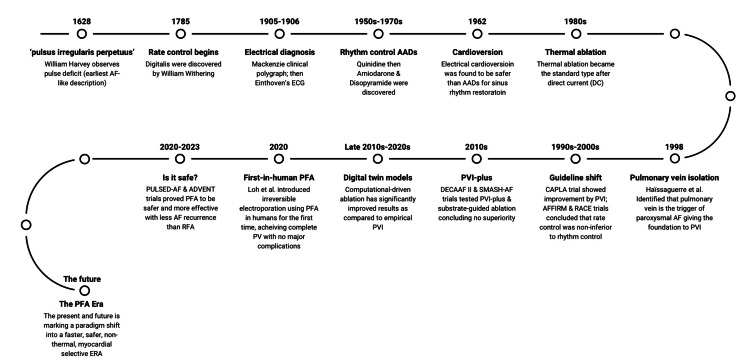
Timeline of atrial fibrillation diagnosis and treatment evolution. Major milestones are shown from Harvey’s 1628 pulse observation to modern pulsed-field ablation (PFA) trials, highlighting breakthroughs in pharmacologic therapy, ablation strategies, and key clinical studies that shaped AF treatment paradigms. Image Credit: Authors

Further advancement led to irreversible electroporation, the mechanism that PFA devices utilize [16,20]. The first-in-human PVI study (2020) demonstrated brief, high-voltage, non-thermal, myocardial-selective pulses that ablate cells without thermal injury, markedly reducing the risk of collateral damage compared with RF or cryo methods [16,20]. In 10 patients treated, all PVs were isolated, and there were no procedural complications [20]. Thus, PFA has emerged as a new, effective, and safe source of energy that has the potential to reshape AF ablation practice [16]. Figure 1 illustrates the timeline of major milestones in AF diagnosis and treatment.

This narrative review aims to synthesize the most current evidence on PFA in the context of first-line catheter ablation for paroxysmal AF, with particular emphasis on its distinctive safety profile and procedural characteristics. Specifically, we summarize data comparing the efficacy, safety, and efficiency of PFA with established thermal ablation modalities; evaluate whether current evidence supports its use as a first-line ablation option in eligible patients; and highlight key controversies, evidence gaps, and priorities for future research that must be addressed before PFA can be considered a routine default modality.

Methodology

We performed a focused literature search on PFA and AF management on PubMed and Google Scholar using "atrial fibrillation", "pulsed field ablation", "catheter ablation", "radiofrequency ablation", "cryoablation", and "first-line therapy" as the keywords. To facilitate the inclusion of studies reflecting historical and present perspectives, no specific date restrictions were imposed. Only peer-reviewed, English studies were selected. Most evidence was collected on patients with paroxysmal AF. Nevertheless, relevant evidence on other subtypes of AF was included. Grey literature and non-peer-reviewed literature were excluded. This review is presented in narrative format and is intended to synthesize the available evidence on PFA in an integrative manner rather than perform a systematic review level of review.

## Review

Effectiveness: ablation vs. drug, and the emergence of PFA

Effectiveness is the most studied outcome in the management of AF. Aiming to maintain sinus rhythm, relieve symptoms, and prevent disease progression [3,25,26]. Historically, AAD therapy was the strategy of choice for rhythm control, and ablation was reserved for the refractory cases [27,28]. Increasing evidence supported the superiority of ablation and led to the classification of ablation as a first-line therapy [3,6,7].

Ablation vs. AADs

Meta-analyses have demonstrated the superiority of thermal ablation (including radiofrequency and cryoballoon) to AAD therapy. One meta-analysis of six randomized controlled trials (RCTs) (1212 patients) showed that ablation therapy resulted in a lower rate of recurrence of AF of 38% and a lower risk of hospitalization of 68%, along with no increase in major adverse effects [29]. Another meta-analysis of 12 RCTs showed a reduction in recurrence and hospitalization rates of 56% [28].

The EARLY-AF (Early Aggressive Invasive Intervention for Atrial Fibrillation) trial, utilizing implantable monitors, demonstrated a significantly reduced progression to persistent AF over three years for those patients undergoing primary cryoablation versus AAD therapy (1.9% vs. 7.4%; hazard ratio (HR) 0.25) [30]. The STOP AF First and Cryo-FIRST (Catheter Cryoablation Versus Antiarrhythmic Drug as First-Line Therapy of Paroxysmal Atrial Fibrillation) trials also demonstrated improved success at 12 months with primary cryoablation (74.6% vs. 45.0%) [14,27]. The MANTRA-PAF (Medical ANtiarrhythmic Treatment or Radiofrequency Ablation in Paroxysmal Atrial Fibrillation) trial showed the superiority of radiofrequency ablation (RFA) to medical therapy at five years [31]. The EAST-AFNET 4 trial pointed out the improvement in cardiovascular outcomes for early rhythm control of atrial fibrillation (either ablation or AAD) vs. usual care [3,13].

These conclusions are substantiated by observational data. In the Optum Clinformatics cohort, the authors observed 48% lower hospitalization rates for arrhythmias in those patients undergoing primary ablative therapy versus AAD therapy [32]. The Cryo Global Registry reported an 87.8% success rate for cryoablation therapy at 12 months, underscoring early procedural value [33].

PFA Effectiveness Compared With Thermal Ablation

PFA has rapidly evolved to clinical application with the development of a modality that creates durable lesions and establishes a positive safety profile. The ADVENT (Randomized Controlled Trial for Pulsed Field Ablation versus Standard of Care Ablation for Paroxysmal Atrial Fibrillation) trial established non-inferior status when compared to thermal ablation with regard to one-year effectiveness against arrhythmia recurrence for paroxysmal AF (73.3% with PFA vs. 71.3% with thermal ablation) [34,35]. In a secondary analysis, patients undergoing PFA had a lower post-procedural arrhythmia burden than those treated with thermal ablation, particularly in those patients with prior Class I/III AAD failure (i.e., 86.0% vs. 71.3%) [35]. The SINGLE SHOT CHAMPION RCT, utilizing implantable monitors, also observed a possible signal of superiority of PFA over cryoballoon therapy; however, these findings should be interpreted in the context of trial design and prespecified endpoints [34,36,37].

Meta-analyses and cohort studies showed mixed but generally favorable results. Some have observed equivalent results with regard to rate of recurrence when utilizing thermal ablation [38], while others (largely observational) have reported lower recurrence rates with PFA [39]. However, in general, across these studies, PFA continues to show better results in terms of procedural efficiency [35,38,40].

Foundational single-arm studies (the PULSED AF (Pulsed Field Ablation to Irreversibly Electroporate Tissue and Treat AF), inspIRE (Study for Treatment of Paroxysmal Atrial Fibrillation by Pulsed Field Ablation System With Irreversible Electroporation), AdmIRE (Assessment of Safety and Effectiveness in Treatment Management of Atrial Fibrillation With the Bosense-Webster Irreversible Electroporation Ablation System), FARA-Freedom trials including drug-refractory patients) report success rates at one year of 66-81% [36-39,41-44], broadly similar to, and in some series numerically higher than, those reported for thermal ablation in prior cohorts [41-43]. One sub-study of the EUropean Real World Outcomes with Pulsed Field AblatiOn in Patients with Symptomatic AtRIAl Fibrillation (EU-PORIA) registry found that patients who were drug refractory did not have a significantly different results as compared to those who had first-line therapy in terms of their rate of recurrence of paroxysmal AF (i.e., 80.3% vs. 78.0%) suggesting that there is no loss of effectiveness with PFA irrespective of the prior drug history of the patients, possibly due to a “ceiling effect” [44-46].

Safety and complications: comparing procedural risks vs. drug side effects

Safety factors are critical in deciding on first-line therapy [3]. The choice between invasive ablation and chronic medical therapy is a balancing act between individual risks associated with the acute procedural choice and cumulative risks associated with the chronic use of medication [6,31]. PFA is designed to weigh this balance toward earlier procedures by diminishing the feared complications of thermal ablation [39,41,43,44].

Thermal Ablation vs. AADs: The Established Balancing Act

RCTs and meta-analyses showed the overall rates of adverse events to be similar with thermal ablation and with AADs, but the nature of adverse events differs [29]. A meta-analysis of six RCTs showed there is no statistically significant difference in composite adverse events, although the rate was slightly higher with ablation (4.2% vs. 2.8%) [29]. Another review showed increased acute procedure-related risk with ablation (risk ratio (RR) 1.51) but no mortality difference [28]. Major procedural complications are infrequent and, should they occur, can be serious, but reported rates vary by era, technology, surveillance intensity, and center volume; cardiac tamponade (RR 9.22 vs. AADs), atrio-esophageal fistula (~ 0.2%, often fatal), stroke/transient ischemic attack (TIA) (0.1-1.0%), persistent phrenic nerve palsy (increased incidence with cryoablation), vascular complications (1-7%) [6,28].

Conversely, chronic toxicities and pro-arrhythmia are associated with AADs [6,47]. Class IC agents are known to increase the risk of sudden cardiac death in structural heart diseases, as seen in PRAGUE-25 [6,48]. The Class III agents cause torsades de pointes, while amiodarone is associated with substantial extra-cardiac (lung, thyroid, liver) toxicity [6]. In EAST-AFNET 4, serious adverse events directly related to rhythm control were increased in the intervention (4.9% vs. 1.4%) but remained infrequent [13]. In real-world data, the first-line use of catheter ablation is associated with decreased rates of hospitalization for both AF and HF and overall cardiac events [32]; likewise, the low event rates were confirmed in patients undergoing first- line cryoablation in the Cryo Global Registry (2.3%) [33].

PFA: A Paradigm Shift in the Safety Model

PFA uses irreversible electroporation, which is non-thermal and selectively ablates cardiomyocytes, while sparing adjacent structures [39,41]. The PULSED AF trial described a primary safety event rate of 0.7%, with no mention of significant problems such as atrio-esophageal fistula, permanent phrenic nerve injury, or PV stenosis [41]. AdmIRE and InspIRE showed extremely low event rates (2.9% and 0%) with no thermal complications [42,43]. Multiple studies, such as ADVENT and FARA-freedom, demonstrated non-inferior safety when compared with thermal ablation and demonstrated superior rates with respect to the prevention of PV narrowing [35,44,49,50]. The large datasets from real-life experience (FRANCE-PFA (FRench Nationwide Cohort of Pulsed Field Ablation), FARADISE, MANIFEST-PF (Multi-National Survey on the Methods, Efficacy, and Safety on the Post-Approval Clinical Use of Pulsed Field Ablation)) have confirmed these results, with significantly low rates of complications (0.96-1.6%) and an absence of esophageal or phrenic nerve injuries [51-53,54]. Meta-analyses consistently show lower rates of phrenic nerve palsy with PFA as opposed to cryoballoon ablation [38,55,56]. The early reports of significantly increased rates of tamponade were likely due to factors associated with the learning curve, as well as delivery system issues unique to the technology and its application (e.g., stiff guidewire), with rates decreasing over time in large registries [38,43,51].

Patient selection: which populations benefit more?

The success of arrhythmia ablation strategies largely depends upon patient selection [47,57]. For PFA, the key is identifying the subgroups by AF type, stage of disease, or comorbidity that derive the most benefit [58]. Evidence is increasingly defining the ideal patients for this technology [46].

Early RCTs established that first-line ablation was better than AADs in younger, symptomatic patients who had paroxysmal AF and minimal structural heart disease [27,29,47,59]. PFA trials (ADVENT, PULSED AF, FARA-Freedom, AdmIRE) confirmed efficacy in the setting of drug-refractory paroxysmal AF [35,41,43,44,60]. A secondary analysis of ADVENT found that PFA was 2.5 times more likely than thermal ablation to achieve a very low (<0.1%) arrhythmia burden with PFA compared to thermal ablation [35]. Similarly, PULSED AF reported higher one-year success in paroxysmal (66.2%) vs persistent AF (55.1%) [41].

Large registries (FARADISE, EU-PORIA) consistently demonstrate paroxysmal AF as the predominant and most treated group, with 80% as opposed to 60-70% for persistent AF for one-year success [52,46]. French real-life data also showed a one-year success rate of 88.4% for paroxysmal AF vs 69.7% for persistent AF [61].

The 2023 ACC/AHA and 2024 ESC guidelines both class I highly recommend first-line catheter ablation in symptomatic paroxysmal AF [6,7]. Meta-analyses show similarly that ablation is better than AADs in both arrhythmia types, but the success is still greater in paroxysmal arrhythmias [29].

Quality of life (QoL) and functional outcomes of different AF treatment strategies

The treatment of AF has a significant impact on various parameters of QoL and functional status in a patient. Catheter ablation results in significant symptom relief and disease burden reduction; however, the amount of relief depends on the type of technology utilized for treatment, timing, patient characteristics, and outcome definitions.

Catheter Ablation vs. AAD Therapy

Comparative trials show consistently more improvement in QoL and functional status after catheter ablation than after AAD therapy, particularly in the first year after the procedure. In the EARLY-AF trial, patients who had cryoballoon ablation had significantly better Atrial Fibrillation Effect on Quality of Life Questionnaire (AFEQT) and EuroQol 5 Dimension (EQ-5D) results than those treated with AADs [30,59]. The CABANA (Catheter Ablation versus Antiarrhythmic Drug Therapy for Atrial Fibrillation) trial has duplicated these results with significant improvement in Atrial Fibrillation Effect on Quality of Life (AFEQT) and Mayo AF-Specific Symptom Inventory (MAFSI) scores in both sexes at 12 months with less hospitalization and AF burden [62,63]. Patients who underwent catheter ablation in these trials reported significantly fewer hospitalizations and AF burden in the long term [30].

The CAPTAF (Catheter Ablation Compared with Pharmacological Therapy for Atrial Fibrillation) trial showed better freedom from arrhythmia with ablation but loss of QoL superiority by 48 months due to a 46% crossover from the AAD group to ablation [57,64]. Notably, CABANA also pointed out the significant fact that the benefit was most notable in those patients with lower baseline QoL, whereas those with mild symptoms (AFEQT ≥ 70) derived very little additional benefit in QoL, particularly in females [62,63].

The Role of Technology and Procedural Technique to Maximize Outcomes

Post-ablation arrhythmia burden is a more robust predictor of QoL and outcomes than standard endpoints of recurrence. In ADVENT, arrhythmia burden > 0.1% correlated with poorer QoL and higher reintervention rates, while PFA achieved superior low-burden outcomes, especially in patients with prior Class I/III AAD failure [35].

Emerging technologies show promising QoL and functional gains. The Q-FFICIENCY trial employing a temperature-controlled catheter demonstrated a 31-point mean AFEQT improvement and 94% reduction in cardioversions [42,65]. Correspondingly, the SmartfIRE trial employing dual energy ablation produced a 26.9-point median AFEQT gain with a 41% reduction in cardiovascular hospitalizations [66]. Adherence to procedural workflows was crucial, as highly adherent patients following procedural workflow demonstrated an 86.9% arrhythmia-free survival at 12 months as opposed to 64% in the low-adherent patient group [66].

Early Ablation and Lifestyle Modification

Early intervention improves rhythm control and patient-reported outcomes. Data derived from the Cryo Global Registry and EARLY-AF demonstrated that first-line cryoablation improved symptoms and reduced persistent AF progression as compared to ablation delayed after AAD failure [33,30,59].

The PRAGUE-25 trial showed that intensive lifestyle modification in addition to AAD therapy resulted in QoL and rhythm outcomes comparable to ablation in obese AF patients [48]. Loss of weight, improvement in glycemic control (HbA1c), and better exercise tolerance (VO₂ max) suggest that lifestyle therapy can be a synergistic rather than a substitute for ablation [48].

Specific and High-Risk Patient Populations Outcomes

Ablation provides a great advantage even in complex patient groups. A meta-analysis demonstrated its superiority to non-ablation therapy in concurrent AF and HF patients, by showing benefit in left ventricular ejection fraction (LVEF), functional capacity (six-minute walk test), New York Heart Association (NYHA) class, and QoL using the Minnesota Living With Heart Failure Questionnaire (MLHFQ) [67].

Moreover, a study conducted by Martins et al. on elderly patients (aged ≥65) using the pulmonary vein ablation catheter (PVAC) gold catheter illustrated that both ablation and AADs improved QoL with an insignificant difference [68]. Surprisingly, Martins et al. concluded that in this older population, there was a 25% incidence of asymptomatic cerebral embolization, highlighting that even the safety profiles of technologies are an important aspect of functional outcomes in high-risk populations [68]. Their investigations were inspired by the findings of the PRECISION GOLD trial, which concluded that the rate of cerebral embolization was only 2.1% [69]. Shockingly, when the methodology of both studies was reviewed, it was clearly stated that the PRECISION GOLD trial performed a cerebral MRI within 48 hours before the procedure and 16-72 hours after the ablation [69]. On the contrary, the PVAC Gold study performed a cerebral MRI 48 hours post ablation, with no pre-ablation baseline imaging for comparison, which makes the 25% incidence finding questionable [68].

Consistent with recent analyses in persistent AF, it was illustrated that recurrence of arrhythmia in paroxysmal rather than persistent AF gave a significantly improved QoL to those patients who underwent these procedures. This highlights that even partial improvement in the rhythm does enhance patients’ functional outcomes [70].

Cost-effectiveness of the different AF treatment strategies

PFA value assessments constantly emphasize the economic value of this approach due to outcomes and efficiency that can balance out the higher upfront costs of a new device. The PFA technology’s cost-effectiveness has also been studied in comparison with thermal ablation technologies.

PFA vs. Thermal Ablation (United States Payer Perspective)

A cost-effectiveness analysis based on the ADVENT trial suggested that, from a United States payer perspective, PFA could be both less costly and slightly more effective than thermal ablation over 5- to 40-year time horizons [35, 71], a pattern often described as ‘dominant’ in health economic terms. In that model, the incremental advantage of PFA was driven by fewer procedure- and AF-related strokes, lower rates of treatment failure, and fewer serious energy-directed adverse events. A budget impact model suggested that the more PFA devices are adopted, the higher the cost savings for payers will be [71].

PFA‘s Procedural Efficiency Leads to Its Cost-Saving (European Hospital Perspective)

A cost-consequence analysis from a European hospital perspective suggested PFA provides significant cost savings simply through better resource consumption, primarily by reducing procedure time [40]. PFA procedures had the shortest procedure time (mean skin-to-skin time of 50.9 minutes), compared to cryoballoon (74.5 minutes) and RFA (140.2 minutes) [40]. As a result of the procedure times, this likely enables hospitals to do more procedures at the same time to optimize hospital utilization, such that it helps decrease waitlists [40]. The one-year horizon use (excluding kit costs) of PFA was estimated to save €850 (~$998)/patient more than a cryoablation kit and €1,301 (~$1528)/patient compared to RFA. In terms of ablation kit costs, the savings achieved through efficiency resulting from PFA can account for the additional price of the device. However, PFA will remain cost-saving even if the kit costs up to €850 (~$998) more than a cryoablation kit, or €1,301 (~$1528) more than an RFA kit [40].

PFA‘s Reduced Repeat Procedures Lead to Its Cost-Saving (United Kingdom NHS Perspective)

A cost analysis based on comparisons in the United Kingdom NHS indicated that PFA was cost-saving compared to cryoablation within 12 months due to improved durability and safety [72]. PFA was approximately 3% less costly (£343 (~$461) savings) than cryoablation in 12 months [72]. Although the first catheter for the PFA is high, the first catheter costs are far more than compensated by additional cost savings as a result of less repeat ablations, as the costs of repeat procedures were more than 50% lower due to lower rates of AF recurrence (15.4% AF recurrences for PFA vs. 32.0% for cryoablation) [72]. In addition, the costs of complications management were close to 50% less for PFA [72].

Guidelines and expert consensus

Despite some methodological variations, all contemporary guidelines and expert consensus are in agreement that PVI is the cornerstone of AF ablation [6,7,73]. Between the old and most recent guidelines, both the American and European societies have promoted the performance of catheter ablation earlier, as it is to be considered first-line therapy for selected patients with symptomatic paroxysmal AF, and more broadly in those with heart failure [6,7,74,75]. Importantly, all of these guidelines intentionally remain agnostic regarding the specific energy source used to achieve pulmonary vein isolation, and do not currently recommend PFA over thermal modalities such as RF or cryoballoon ablation.

The techniques of RF and cryoballoon ablation remain established with similar efficacy, while PFA has developed as an attractive non-thermal alternative with good safety profiles, mounting evidence of non- inferiority [7,73]. Lesions outside those of PVI, including posterior wall isolation, rotor mapping, CFAE ablation, or fibrosis-guided strategies, have shown inconsistency or marginal benefit in randomized trials and are not generally recommended [6,73]. Hybrid approaches to ablation and surgical ablation may be considered in selected patients who have long-standing persistent AF or who may have surgical procedures, but should be performed based on institutional expertise and shared decision making [7].

The 2023 ACC/AHA/American College of Clinical Pharmacy (ACCP)/Heart Rhythm Society (HRS) guideline constitutes a major update with the emphasis on earlier rhythm control and allocating catheter ablation the designation of Class I for symptomatic and Class IIb for asymptomatic paroxysmal AF. It also emphasizes oral anticoagulation uninterrupted for at least three months after ablation and discourages the pursuit of extra-PVI targets routinely [6].

The 2024 ESC guideline updates the “AF-CARE” framework, prioritizing comorbidity management and recommending ablation as one of the first-line options for symptomatic paroxysmal AF (Class I). For persistent AF, catheter ablation is considered Class IIb with rhythm control or ablation encouraged within 12 months of diagnosis [7].

The 2024 European Heart Rhythm Association/HRS/Asia Pacific Heart Rhythm Society/Latin American Heart Rhythm Society expert consensus statement on catheter and surgical ablation of AF considers technical execution requiring complete PV isolation and gives detailed metrics for procedures (contact force, ablation index, first-pass isolation, high-power short-duration radiofrequency, cryo dosing). It recommends the use of PFA with its favorable safety and efficiency, and treatment delay increases recurrence by approximately 20%, and emphasizes that neither adenosine nor waiting tests are necessary with current systems [73]. To provide a concise comparison of these recommendations, Table 1 summarizes the key differences among the most recent American, European, and International guidelines and expert consensus documents on atrial fibrillation management.

**Table 1:** The differences between the most recent American, European guidelines and the international consensus Summarizes variations in framework, classification, risk scoring, and ablation guidance among the 2023 ACC/AHA/ACCP/HRS, 2024 ESC, and 2024 International Ablation Consensus Statements. AF: atrial fibrillation; LRFM: lifestyle and risk factor modification; CHA₂DS₂-VASc: Congestive heart failure, Hypertension, Age ≥75 (doubled), Diabetes, Stroke/TIA (doubled), Vascular disease, Age 65-74, and Scx (female); ESC: European Society of Cardiology; PWI: posterior wall isolation; CFAE: complex fractionated atrial electrograms; ACC: American College of Cardiology; AHA: American Heart Association; ACCP: American College of Clinical Pharmacy; HRS: Heart Rhythm Society

Comparison Area	2023 ACC/AHA/ACCP/HRS Guideline [6]	2024 ESC Guideline [7]	2024 International Ablation Consensus [73]
Core Framework	AF Staging (1-4) and 3 Pillars of Management built on LRFM. Focuses on AF as a progressive disease continuum.	AF-CARE Framework, which explicitly prioritizes [C] Comorbidity management as the initial step for all patients.	HEAD2TOES Schema for pre-procedural risk factor optimization specifically to improve ablation outcomes.
AF Classification	Introduces a new staging system (Stages 1-4) to complement the traditional duration-based classification.	Adheres to the traditional temporal classification (Paroxysmal, Persistent, etc.).	
Stroke Risk Score	Recommends validated scores like CHA₂DS₂-VASc for risk stratification. ESC excludes sex from the score.		
Ablation Guidance	Gives high-level recommendations, establishing ablation as first-line therapy (Class 1) and PVI as the primary technique.	Provides similar high-level recommendations for first-line ablation in paroxysmal AF (Class I).	Provides extensive, granular detail on procedural specifics, such as the futility of CFAE ablation and lack of benefit from routine PWI. It also clarifies that a waiting phase or adenosine test post-PVI is no longer necessary with contemporary technology.

Rationale for considering PFA as a first-line ablation option

Clinical Effectiveness

The importance of considering patient-centered benefits in the decision-making of the therapeutic approach to any disease is non-negotiable. The clinical effectiveness of different treatment approaches is one of the patient-centered benefits.

PFA, however, is an emerging technology that uses non-thermal irreversible electroporation to achieve therapeutic goals, and its efficacy has been studied extensively lately. Across multiple trials, PFA has demonstrated at least comparable freedom from arrhythmia recurrence, with signals of superiority in post- ablation AF burden in certain analyses, especially in class I/III AADs resistance [30-32]. Other studies have found that the one-year success rate of PFA was 66-81%, which is at least comparable to thermal ablation historically [36-39].

Safety Profile and Good Outcome

As outlined previously, both catheter ablation and AAD therapy carry important but different risks: ablation is associated with low but non-trivial rates of acute procedural complications, whereas AADs are limited by long-term toxicities and pro-arrhythmic potential [6,28,29].

When compared to thermal ablation, PFA, on the contrary, was associated with markedly reduced collateral injury, in keeping with its selective irreversible electroporation mechanism. It was designed to be selectively cytotoxic to cardiomyocytes while sparing adjacent non-myocardial tissues like the esophagus, phrenic nerve, and vascular smooth muscle [39,41,49]. Proving its intended consistency, Multiple trials highlighted that PFA had a major complication rate of 0.96-2.9% only, was superior to thermal ablation in preventing PV stenosis, and had 0% occurrence rate of the classical thermal-ablation-related complications [35,36-38,44-47].

QoL

Catheter ablation consistently improves AF-related symptoms and QoL compared with AAD therapy, particularly when used earlier in the disease course [30,57,62]. Early ablation reduces AF burden and progression, and these improvements in rhythm control are closely linked with better patient-reported outcomes [30,33].

Within this context, PFA’s ability to achieve a very low post-ablation arrhythmia burden, such as ≤0.1% in secondary analyses of ADVENT [35], is clinically meaningful, given the strong association between arrhythmia burden, repeat procedures, and QoL measures. Moreover, ablation-based strategies, including those using PFA, remain important even in high-risk populations such as patients with concurrent AF and heart failure, in whom improvements in functional status and QoL have been demonstrated [67,70].

Physicians and Hospital Parameters

From a systems perspective, the choice of ablation technology also affects procedure time, resource utilization, and long-term costs. As detailed in previous sections, economic modeling across different healthcare systems suggests that PFA may be cost-saving or cost-effective compared with established thermal ablation modalities, driven by shorter procedure times, reduced repeat ablation rates, and fewer energy-related complications [40,71,74]. These factors can translate into higher procedural throughput, shorter waitlists, and potential budgetary advantages for hospitals and payers, particularly in high-volume centres with appropriate infrastructure and experience.

The future first-line

On the weight of contemporary evidence, PFA can reasonably be considered one of several first-line catheter ablation options for selected patients with symptomatic paroxysmal AF. The selection of patients, as suggested by the literature, should be younger, symptomatic patients with paroxysmal AF and non-significant structural heart disease [27,29,47,59]. Also, drug-resistant paroxysmal AF patients are excellent candidates for PFA [41,43,44,60], especially Class I/III AADs [35].

This patient selection also goes in parallel with the most recent 2023 ACC/AHA and 2024 ESC guidelines that give the strongest recommendation to catheter ablation (regardless of the energy source) as a first-line therapy in symptomatic paroxysmal AF and AAD-refractory patients (class I evidence) [6,7].

The objective of this review was to explore PFA’s potential role as a first-line therapy in the same population that is now recommended to undergo any catheter ablation in the newest guidelines. While this review provides a comprehensive up-to-date synthesis of current evidence using multiple study designs, its narrative nature inherently limits reproducibility and may be subject to selection bias. Furthermore, the absence of formal quality appraisal and quantitative synthesis means that conclusions should be interpreted with caution. It is imperative to conduct future RCTs and systematic reviews testing the implications of this study.

## Conclusions

PFA has been shown to provide at least non-inferior efficacy compared to traditional thermal ablation options for symptomatic paroxysmal AF while significantly lowering the risk of collateral damage to surrounding tissues and providing better procedural efficiency.

As early catheter ablation is becoming increasingly favored based on both clinical and economic reasons, PFA’s advantages of lower collateral damage and improved procedural efficiency place it as a particularly attractive first-line ablation option for appropriately selected patients and experienced centers. However, current PFA data are limited by shorter follow-up times, selectively enrolled trial populations, and device-specific experiences. Longer-term durability data and comparative evaluations of PFA systems and patient subgroups in real-world studies will be necessary prior to considering PFA a standard energy source for routine practice. 
